# Surface Display of Antigen Protein VP8* of Porcine Rotavirus on *Bacillus Subtilis* Spores Using CotB as a Fusion Partner

**DOI:** 10.3390/molecules24203793

**Published:** 2019-10-22

**Authors:** Wanqiang Li, Jie Feng, Jiajun Li, Jianzhen Li, Zhenhua Wang, Abdul Khalique, Miao Yang, Xueqin Ni, Dong Zeng, Dongmei Zhang, Bo Jing, Qihui Luo, Kangcheng Pan

**Affiliations:** 1 College of Veterinary Medicine, Sichuan Agricultural University, Chengdu 611130, China; LWQ135253@foxmail.com (W.L.); fengjie9201@163.com (J.F.); 18227585635@163.com (J.L.); jianzhenli2006@163.com (J.L.); abdulkhalique36@gmail.com (A.K.); xueqinni@foxmail.com (X.N.); zend@sicau.edu.cn (D.Z.); zdmcypts@163.com (D.Z.); jingbooo@163.com (B.J.); lqhbiology@163.com (Q.L.); 2 Chengdu Vocational College of Agricultural Science and Technology, Chengdu 611100, China; w102672843@163.com; 3 Technology Centre of Chengdu Custom, Chengdu 611100, China; 13548002947@163.com

**Keywords:** porcine rotavirus, VP8* protein, *Bacillus subtilis*, spore surface display, oral immunization, immune responses

## Abstract

Porcine rotavirus is a major cause of acute viral gastroenteritis in suckling piglets, and vaccination is considered to be an effective measure to control these infections. The development of a live mucosal vaccine using *Bacillus subtilis* spores as an antigen delivery vehicle is a convenient and attractive vaccination strategy against porcine rotavirus. In this study, a shuttle vector was constructed for the spore surface display of the spike protein VP8* from porcine rotavirus (the genotype was G5P[7]). A successful display of the CotB-VP8* fusion protein on the spore surface was confirmed by Western blot and immunofluorescence microscopy analysis. The capacity for immune response generated after immunization with the recombinant strain was evaluated in a mouse model. The intestinal fecal IgA and serum IgG were detected by enzyme-linked-immunosorbent serologic assay (ELISA). Importantly, recombinant strain spores could elicit strong specific mucosal and humoral immune responses. These encouraging results suggest that recombinant *B. subtilis* BV could provide a strategy for a potential novel application approach to the development of a new and safe mucosal subunit vaccine against porcine rotavirus.

## 1. Introduction

Rotaviruses are members of the *Reoviridae* family of double-stranded RNA viruses. The virus genome is composed of 11 segments encoding six structural viral proteins and six nonstructural proteins [1,2,3]. Rotavirus is classified into multiple groups by the inner capsid protein (VP6) and the outer capsid proteins (VP4 and VP7), which form the base of the G and *p* dual typing system [4,5]. The main symptom of porcine rotavirus is severe diarrhea, which results in huge economic losses in the pig industry worldwide [6]. Pigs of all ages can be infected with rotavirus, and nursing piglets have more severe symptoms. Infection of weaned piglets is characterized by mild to moderate or no clinical manifestations, but they can continue to be exposed to infectious viruses in the environment [7,8,9]. The virus is transmitted by the fecal–oral route and can survive in the environment for a long time [10]. Therefore, once contaminated, rotaviruses in swineherds are difficult to eliminate. Vaccination is considered to be an effective measure to control these infections. A large vaccine dose of inactivated vaccines is usually required to induce an efficient immunity. An attenuated live vaccine has the excellent property of inducing humoral and cellular responses, but there are certain disadvantages, such as residual virulence and potential infection or spread [11,12]. To overcome these shortcomings, the potential development of a mucosal subunit vaccine expressed in a live vector to deliver a heterologous antigen to the mucosal immune system based on *Bacillus subtilis* spores may be regarded as a promising approach. The virus VP8* protein, cleavage production of VP4 and containing most of the epitopes of VP4, which is linear and relatively conservative, is related to attachment and efficient cell entry, and it can induce neutralizing antibodies that can protect against infection or diseases related to rotavirus [13,14,15,16,17]. It has been indicated that VP8* protein is a promising molecule for use as a subunit vaccine candidate.

*B. subtilis* is a Gram-positive bacterium that can be induced to produce spores when it encounters harsh conditions. *B. subtilis*, which is a generally recognized as safe (GRAS) strain, is widely consumed as probiotics in humans and animals [18]. *B. subtilis* spores possess stability, adjuvant properties, and the ability to transit across the gastrointestinal track and interact with immune cells [19,20,21,22]. It has been confirmed that spore coat protein can be used as a fusion partner for expression and display of vaccine antigens on the spore surface, and recombinant *B. subtilis* spores could elicit protective systemic and mucosal immune responses without an adjuvant [23,24]. Their positive effects, especially the application of spore surface display systems, make them attractive as a superior delivery vehicle for mucosal vaccines.

In this study, we constructed a recombinant strain with a spore surface displaying the heterologous antigen protein VP8* of porcine rotavirus and evaluated its immunogenicity. The aim was to develop an alternative porcine rotavirus mucosal subunit vaccine candidate against rotavirus infection for use worldwide.

## 2. Results

### 2.1. Expression of the VP8* Protein in *Escherichia coli* and the Antiserum

**Figure 1:**
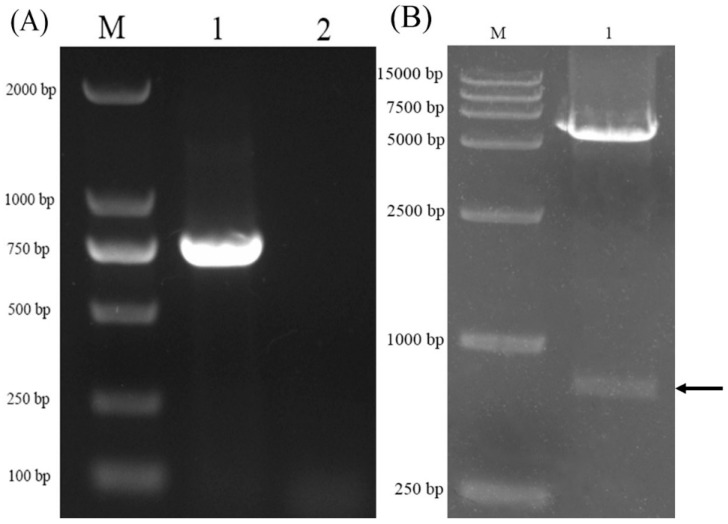
Electrophoretogram of the target gene. (**A**) PCR identification of recombinant *Escherichia coli*; M: DL2000; lane 1: *E. coli* (pET-32a-VP8*); lane 2: *E. coli* Rosetta (DE3). (**B**) PCR identification of pET-32a-VP8*; M: DL15000; lane 1: the products of pET-32a-VP8* by double-restriction-enzyme digestion.

The VP8* DNA fragment of porcine rotavirus G5P[7] was linked to plasmid pET-32a, thus obtaining a prokaryotic expression plasmid pET-32a-VP8*. Recombinant plasmid pET-32a-VP8* was transformed into an *E. coli* Rosetta (BE3) competent cell, and recombinant *E. coli* (pET-32a-VP8*) was amplified and cultured to extract plasmids. Prokaryotic expression plasmids were double-digested by *Bam*H I and *Eco*R I, and the products were identified by agarose electrophoresis. Electrophoresis and sequencing results showed that the VP8* gene of porcine rotavirus was successfully inserted into the prokaryotic expression vector pET-32a, and the prokaryotic expression plasmid pET-32a-VP8* was successfully constructed (Figure 1).

**Figure 2:**
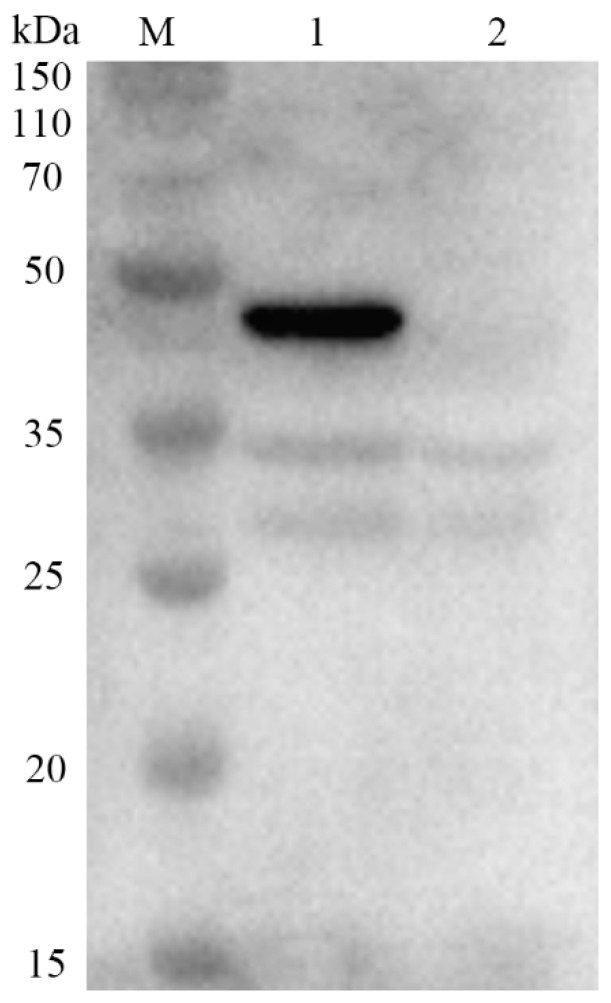
Western blot analysis: Specificity of VP8* protein detected by antirotavirus antiserum. M: protein marks; lane 1: crude cell extract *E. coli* expressing recombinant VP8* proteins; lane 2: protein expressed with the empty pET32a plasmid vector.

After the fourth immunization, the serum of the mice was assayed by ELISA and the antibody titer was 1:12800. The specificity of *E. coli*-expressed VP8* was determined by rotavirus-specific antiserum. Western blot analysis was performed on the total protein from *E. coli* (pET-32a-VP8*) expressing recombinant VP8* protein. The results showed that a protein blot (Figure 2) appeared at the expected size of 45 kDa, which proved that the target protein was successfully induced. It also proved that the serum produced antibodies against porcine rotavirus VP8* protein.

### 2.2. Construction of the Shuttle Vector pDG364-CotB-VP8*

**Figure 3:**
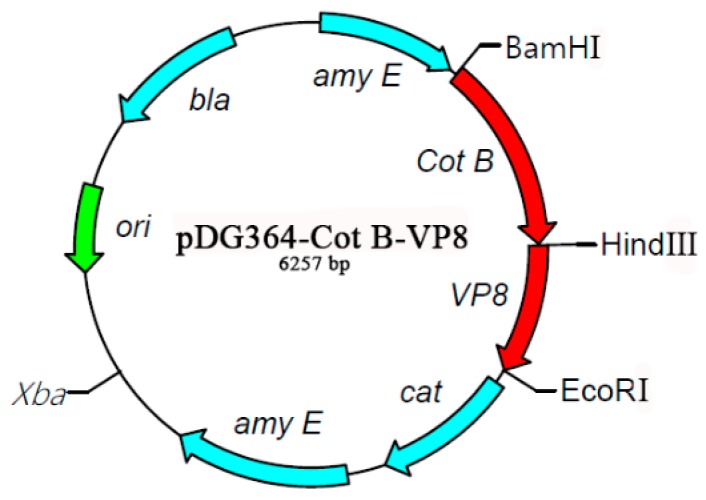
Map of the shuttle vector pDG364-CotB-VP8*. *CotB* and VP8* genes were integrated in the gene fusion on the pDG364 vector.

**Figure 4:**
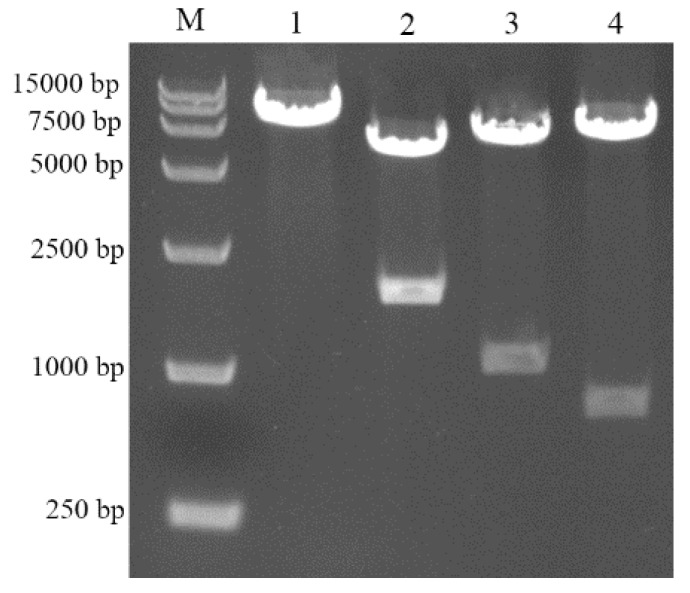
Agarose gel electrophoresis analysis of double-restriction-enzyme digested plasmid pDG364-CotB-VP8*. M: DNA marker; lane 1–4: products from recombinant plasmid digested with Xba I, BamH I/EcoR I, BamH I/Hind III, and Hind III/EcoR I.

The shuttle vector pDG364-CotB-VP8* was constructed on the foundation of the plasmid pDG364 (Figure 3). When protein *CotB* was used as a carrier protein, only the DNA encoding the N-terminal 275 amino acids was used. The coding part of VP8* was fused in frame to the coding part of *CotB*, as specified below. The vector was identified by double-enzyme digestion (Figure 4). These results indicated the occurrence of correct chromosomal integration of CotB-VP8* gene fusion.

### 2.3. Chromosomal Integration of Gene Fusion

**Figure 5:**
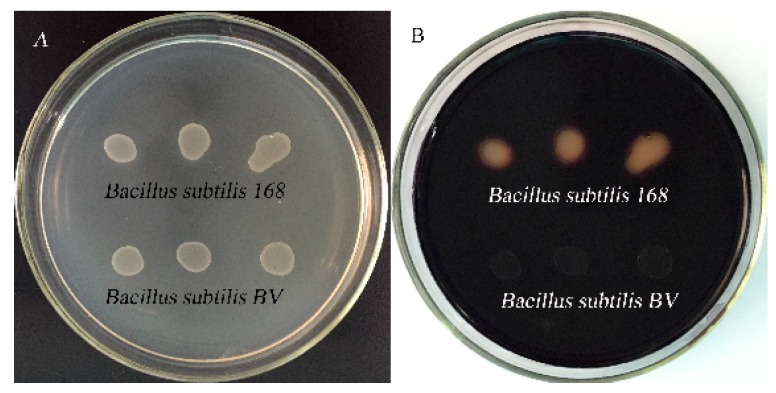
Starch hydrolysis test. (**A**) *Bacillus subtilis* 168 and recombinant *B. subtilis* BV cultivate on medium containing 1% starch. (**B**) The plate stained with iodine.

**Figure 6:**
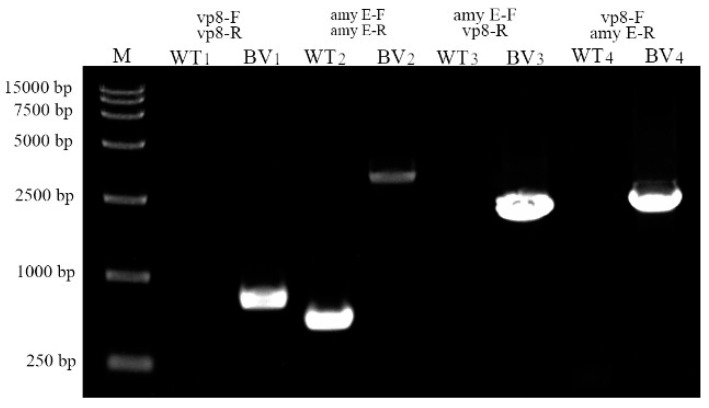
PCR analysis with different primer pairs. M: DNA marker. The PCR primers were as follows: (1) VP8*-F and VP8*-R; (2) amyE-F and amyE-R; (3) amyE-F and VP8*-R; (4) VP8*-F and amyE-R. The PCR template is as follows: (WT) wild-type (*B. subtilis* 168); (BV) recombinant *B. subtilis* BV.

Due to homologous double-crossover recombination, the exogenous fusion gene was inserted into the *amyE* gene of the *B. subtilis* chromosome, resulting in the inactivation of the *B. subtilis* amylase gene. As a result, the recombinant bacteria could not enzymatically hydrolyze the starch and there were no hydrolysis circles around the recombinant bacteria stained with iodine solution, while hydrolyzed circles appeared around the wild-type *B. subtilis* 168 strain (Figure 5). To further verify whether the fusion gene was correctly integrated into the genome of *B. subtilis* 168, PCR identification was conducted with multiple pairs of primers. Electrophoresis of PCR products showed the presence of target bands in the theoretical value area (Figure 6). Meanwhile, sequencing of PCR products showed that the fusion gene was correctly integrated into the *amyE* gene locus of the *B. subtilis* 168 genome.

### 2.4. Determination of Fusion Protein Expression on the *B. Subtilis* Spore Surface

**Figure 7:**
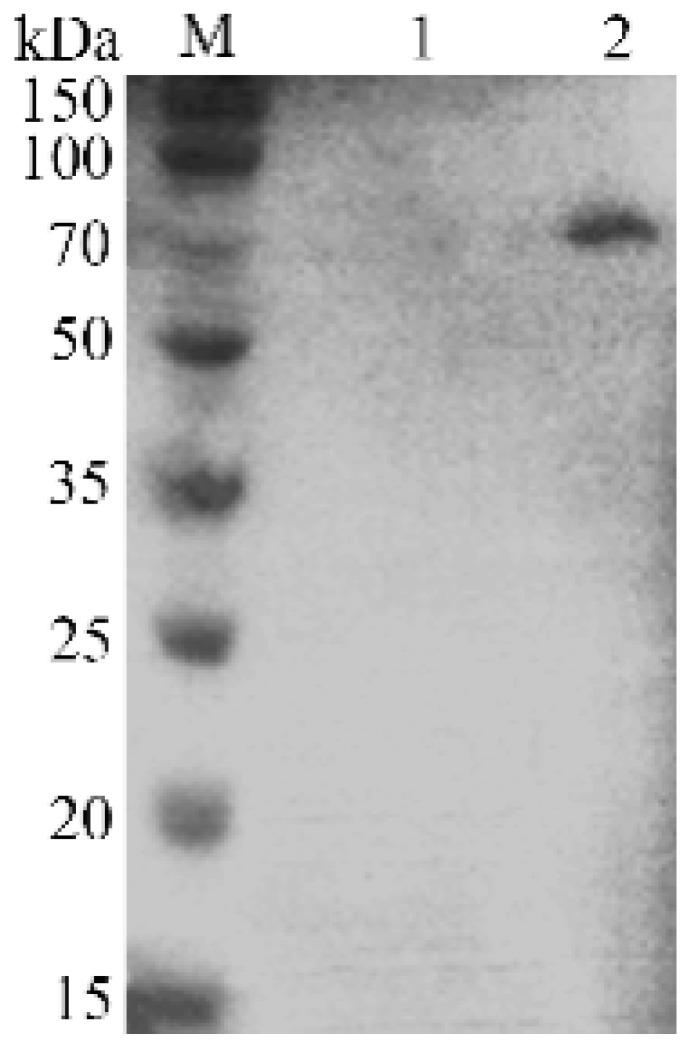
Western blot analysis of spore coat protein. M: TureColor prestained protein marker; lane 1: the spore coat protein of *B. subtilis* 168; lane 2: the spore coat protein of recombinant *B. subtilis* BV.

To verify whether VP8* can be expressed correctly during the formation of spores in recombinant bacteria, the spore capsid protein was extracted for Western blotting analysis. The expression of fusion protein CotB-VP8* was confirmed by Western blotting with a previously prepared rotavirus-specific polyclonal mouse antiserum. A specific protein band of about 70 kDa was found in recombinant *B. subtilis* BV, while none was found in *B. subtilis* 168 (Figure 7).

### 2.5. Immunofluorescence Microscopy Analysis

**Figure 8:**
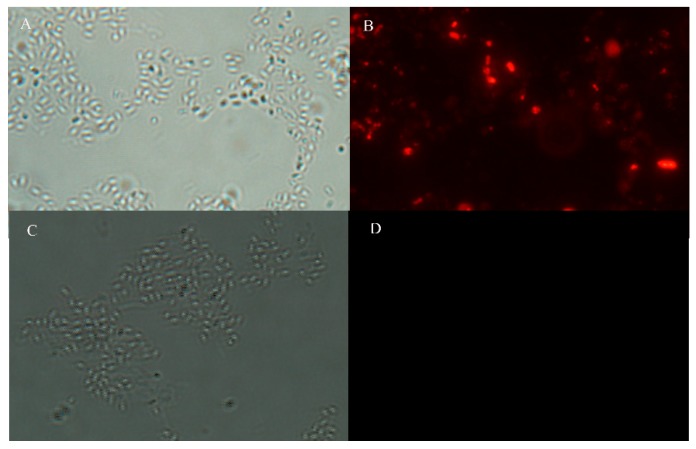
Immunofluorescence microscopy analysis: (**A**,**B**) recombinant *B. subtilis* BV; (**C**,**D**) *B. subtilis* 168; (**A**,**C**) bright-field images; (**B**,**D**) immunofluorescent images (10 × 100).

To detect the surface expression of the fusion protein, sporulating cells of recombinant and wild-type strains were analyzed by immunofluorescence microscopy. A strong fluorescence signal was observed around recombinant spores, while the wild type was negative (Figure 8). Western blotting and fluorescence microscopy showed that the fusion gene CotB-VP8* could be expressed at the promoter of the *CotB* gene when recombinant *B. subtilis* formed spores.

### 2.6. Oral Delivery of the VP8* Protein Displayed on Spores Induced Serum and Mucosal Antibody Responses

**Figure 9:**
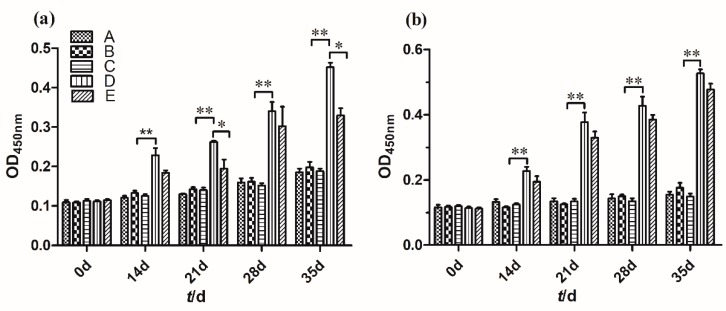
Recombinant *B. subtilis* BV spores induced specific mucosal and systemic immune responses following oral administration. The control group (A) was fed with a basal diet. B and D were fed a diet mixed with *B. subtilis* 168 and recombinant *B. subtilis* BV, respectively. C and E were orally immunized by gavage with *B. subtilis* 168 and recombinant *B. subtilis* BV. The feces and serum collected from mice on days 0, 14, 21, 28, and 35 were analyzed for rotavirus-specific (**a**) IgA and (**b**) IgG (the value of OD_450nm_ (Optical density at 450 nm) was calculated) separately by ELISA. *, *p* < 0.05; **, *p* < 0.01. The error bars represent standard deviations.

Rotavirus-specific fecal IgA and serum IgG antibodies were analyzed by ELISA to evaluate the ability of recombinant spores to elicit mucosal and systemic immunity, respectively. As shown in Figure 9, Table 1 and Table 2, from 14 to 35 days, specific IgA levels (a) in feces of mice with two administered methods induced by *B. subtilis* BV spores were increased compared with *B. subtilis* 168 (*p* < 0.01). The antibody levels in the group fed a diet mixed with 5.0 × 10^6^ CFU/g spores of recombinant *B. subtilis* BV was higher than that in the group orally received immunized spores on days 21 and 35 (p < 0.05). From 14 to 35 days, the serum IgG antibodies (b) elicited by *B. subtilis* BV were similar to each other but higher than *B. subtilis* 168 (*p* < 0.01). The findings indicated that recombinant *B. subtilis* BV can could elicit antibody production in mice. Moreover, mice dosed by being fed a diet mixed with recombinant spores presented significant levels of anti-VP8* serum IgG and intestinal mucosal SIgA responses compared with mice dosed by gavage with the same strains.

## 3. Discussion

In this study, we constructed *B. subtilis* BV that surface displayed the VP8* protein on the spore using CotB as a carrier, which may be an alternative porcine rotavirus mucosal subunit vaccine candidate. The fusion protein CotB-VP8* could maintain its immunogenicity on the spore. It was confirmed that the recombinant *B. subtilis* BV spore was able to deliver the VP8* antigen protein to intestinal mucosa by the oral administration route and it induced both mucosal (IgA) and systemic (IgG) immune responses.

The mucous membranes are one of the largest organs of the body. Mucosal immune lymphoid organs and immunocompetent cells within the mucosal-associated lymphoid tissues (MALT) together constitute a complete mucosal immune system network for the simultaneous induction of IgA responses, which is a strong barrier to the invasion of pathogenic microorganisms and enteric toxins [25,26]. Rotavirus invades the intestine first, mainly inducing an intestinal mucosal immune response. When the intestine is infected, the mucosal immune system network not only elicits an immune response in the mucosa and other mucosal tissues but also triggers a systemic humoral immune response [27,28]. The mucosa is a candidate site for vaccination because it can provide immune protection at the earliest stages of pathogen invasion. Also, this method offers some practical advantages, such as being low cost, the ease of widespread immunization, and the avoidance of injections to reduce the risk of transmission of bloodborne diseases [29,30].

The strategy of spore surface display is generally based on a spore coat protein acting as a carrier, which can anchor a heterologous vector protein on the spore surface [31]. Many coat proteins can be used as a carrier, such as *CotB*, *CotC*, *CotG*, *CotE*, *OxdD*, and so on [32,33,34,35,36]. Previous studies have reported that *B. subtilis* spores could germinate and repopulate in the gut lumen and have beneficial effects on strengthening the immune system [37,38]. It has also been shown that recombinant *B. subtilis* spores could recruit more DCs (Dendritic cells) into the intestinal epithelium, and the recruited DCs could sample spores and bring the labeled spores to mesenteric lymph nodes [39]. Most IgA^+^ plasmablasts are generated with the help of T cells, and the adjuvant activity of *B. subtilis* increasing T-cell responses may be beneficial to broadening the IgA antibody [40]. These studies suggest that spores of recombinant *B. subtilis* BV should be an excellent vehicle for a mucosal subunit vaccine. Spore-based vaccines are also safe, stable, low cost, and conveniently administered.

Rotavirus, as a common intestinal pathogen, causes acute gastroenteritis in young children and animals. The ability to provide protection from homotypic rotavirus infection depends on whether the level of circulating serum antibody is high enough, as the protection is limited if the level of serum antibody is not sufficiently high [41,42,43]. This phenomenon may be associated with the ability to induce antibody transfer into the gut lumen. Previous studies have suggested that the immune effector functions of mucosal antibody titers or levels of virus-specific memory B cells in the gut appear to be more protective against rotavirus infection and illness than the serum antibody levels [44,45,46]. For this reason, most efforts so far have focused on orally administered live attenuated vaccines. Several live attenuated vaccines that are administered orally have been licensed for human use [47]. However, one does not exist for veterinary use. Inactivated and attenuated live vaccines are widely used in herds with injection [11]. Most genotypes of porcine rotavirus strains are OSU-like (*p*[7]) or Gottfried-like (*p*[6]), isolated from continents. The single most prevalent genotype combination is G5P[7] in swine herd [48]. So, we chose the porcine rotavirus with genotype G5P[7] as the target strain. The VP8* protein was successfully displayed on the spore. Recombinant *B. subtilis* BV spores elicited both mucosal (IgA) and systemic (IgG) immune responses by oral administration. These results suggest that *B. subtilis* BV could be used as a potential vaccine against rotavirus infection in pigs.

## 4. Materials and Methods

### 4.1. Plasmids, Strains, and Primers Sequences

The bacterial strains and plasmids used in this study are listed in Table 3. *E. coli* and *B. subtilis* 168 strains were grown in LB medium at 37 °C. Antibiotics were added as selective agents when needed: ampicillin (100 μg/mL) for *E. coli* and chloramphenicol (5 μg/mL) for *B. subtilis*.

### 4.2. Expression of the VP8* Protein in *E. coli* and Antiserum Production

The prokaryotic expression vector pET32a-VP8* was constructed and transformed into *E. coli* Rosetta (BE3). The recombinant protein was expressed by induction with isopropyl-β-D-thiogalactoside (IPTG) at a final concentration of 1 mM when absorbance at 600 nm reached 0.5. After induction for 3 h at 37 °C, cells were harvested by centrifugation at 10,000× *g* for 15 min at 4 °C [49]. Proteins were separated on a 12% SDS-PAGE gel and transferred onto a PVDF membrane. The membrane was blocked with TBS containing 5% bovine serum albumin (BSA) at 37 °C for 2 h, washed five times with TBST, and incubated with mouse antirotavirus serum at 4 °C overnight. Following washing five times, the membrane was incubated with an HRP-conjugated goat anti-mouse IgG for 1 h at room temperature. The blot filters were visualized by the ECL Western Blot Detection Kit as specified by the manufacturer [50].

Porcine rotavirus vaccine was administered intraperitoneally to BALB/c mouse (20 g), followed by three boosts at 7-day intervals. Blood samples were collected on day 3 after the last injection. The antiserum was pooled together and stored at −80 °C [51]. The titers were determined by enzyme-linked-immunosorbent serologic assay (ELISA). All animal experiments were performed in accordance with the guidelines for the care and use of laboratory animals and approved by the Institutional Animal Care and Use Committee of Sichuan Agricultural University (approval number: DY2018203046).

### 4.3. Plasmids and Strain Construction

An *E.*-*coli*–*B.*-*subtilis* shuttle vector (pDG364-CotB-VP8*) was constructed for surface display of VP8* on the spores of *B. subtilis*. Both the purified VP8* fragment and the *E.*-*coli*–*B.*-*subtilis* shuttle vector pDG364 were double-digested by Hind III and EcoR I, and the VP8* was ligated into the shuttle vector pDG364. The pDG364-VP8* recombinant vector was obtained. Using the same method to digest the CotB fragment and pDG364-VP8* by BamH I and Hind III, we obtained the pDG364-CotB-VP8*. The integrity and fidelity of the fragment was confirmed by agarose gel electrophoresis analysis of double-restriction-enzyme digestion and DNA sequencing.

Plasmid pDG364-CotB-VP8* was linearized by digestion with Xba I and used to transform competent cells of *B. subtilis* 168. Cm^r^ clones were the result of double-crossover recombination. All Cm^r^ clones were tested for amylase activity. The amylase-inactivated strain was correctly integrated in the interruption of the nonessential *amyE* gene on the *B. subtilis* chromosome. Chromosomal DNA extracted from Cm^r^ clones and the amylase-inactivated strain were used for PCR identification with several different pairs of primers (Table 3).

### 4.4. Preparation of Spores

Recombinant *B. subtilis* BV was cultivated in Difco sporulation medium (DSM). After 40 h of incubation at 37 °C, spores were collected, washed several times, and purified by lysozyme treatment, as previously described [32]. The number of purified spores was measured by the flat colony counting method. *B. subtilis* 168 was induced to spore by the same method. The spores were stored at −20 °C until used.

### 4.5. Western Blot Analysis

Spore coat proteins extracted from the purified spores of recombinant *B. subtilis* BV were processed by sodium dodecyl sulfate (SDS)-dithiothreitol (DTT) treatment at 65 °C for 10 min [52], and the protein solution was concentrated by freeze-drying. Concentrated proteins were fractionated on 12% denaturing polyacrylamide gels, electrotransferred onto a PVDF membrane, and used for Western blot analysis by standard procedures. Western blot filters were visualized by the enhanced chemiluminescence method as specified by the manufacturer.

### 4.6. Immunofluorescence Microscopy

Immunofluorescence was used to analyze VP8* protein surface expression on the spores by pDG364-CotB-VP8*, as previously described [52]. Briefly, samples were blocked with PBS 5% BSA for 30 min at 37 °C, washed 5 times in PBST, incubated with mouse anti-VP8* serum for 2 h at 37 °C, washed 5 times, and then incubated further for 1 h at 37 °C in the dark with Cy3-conjugated goat anti-mouse IgG (1:200). After washing five times with PBST, the precipitate was resuspended in PBS, airdried on two glass slides separately, and viewed under an OLYMPUS BX43 fluorescence microscope (Eclipse, TE2000U, Nikon). *B. subtilis* 168 strains were used as negative controls.

### 4.7. Immunization of Mice and Sample Collection

Female mice (20 g) were fed twice daily with a basal diet for 7 days before the administration. They were randomly divided into five groups (25 mice in each group). As shown in Table 4, the control group (A) was fed with a basal diet. Two groups were separately fed a diet mixed with 5.0 × 10^6^ CFU/g spores of *B. subtilis* 168 (B) and recombinant *B. subtilis* BV (D). Groups C and E were orally immunized by intragastric gavage containing 2.0 × 10^10^ CFU/mL of spores in a volume of 0.15 mL on days 1–3, 14–16, and 28–30. Serum and fecal sample were collected on days 0, 14, 21, 28, and 35.

### 4.8. ELISA of Specific Antibodies in Gut and Serum

Mouse fecal IgA and serum IgG were collected at 0, 14, 21, 28, and 35 days for specific anti-VP8* antibodies by ELISA. All samples were stored at −70 °C until assayed by ELISA. Polystyrene microtiter plates were coated with porcine rotavirus overnight at 4 °C and washed five times with PBS 1% Tween 20. After blocking with PBS 5% skimmed milk at 37 °C for 2 h, the plates were washed five times. The fecal wash sample and serum were diluted 1:100 in PBS 0.5%, incubated at 37 °C for 1.5 h, and washed five times. HRP-conjugated goat anti-mouse IgA (1:100) and HRP-conjugated goat anti-mouse IgG (1:2000) were separately added to the ELISA plates. The solution was incubated at 37 °C for 1 h. After washing five times as usual, 100 μL of tetramethylbenzidine was added to each well and reacted at room temperature for 10–15 min. The reaction was stopped by 50 μL of 2 M H_2_SO_4_ and, finally, the optical density value was detected at 450 nm.

### 4.9. Statistical Analysis

Statistical significance was determined using ANOVA, with a *p*-value < 0.05 considered as significant.

## 5. Conclusion

In summary, porcine rotavirus VP8* could efficiently display on the surface of recombinant *B. subtilis* BV with biological activation, and its spores could elicit both special and systemic immune responses in mice. The next step is to test the efficacy of this vaccine formulation in a porcine vaccination and infection model. Additionally, as a promising live subunit vaccine candidate, the subtle potential impact of spores that are excreted into the environment on the possible existence of a microecology should also be evaluated.

## Figures and Tables

**Table 1:** The OD_450_ of serum IgG levels before and after immunizations.

Time			Group		
(d)	A	B	C	D	E
0	0.1151 ± 0.0149 ^a^	0.1169 ± 0.0063 ^a^	0.1192 ± 0.006 ^a^	0.1132 ± 0.0087 ^a^	0.112 ± 0.0065 ^a^
14	0.1328 ± 0.014 ^B^	0.1164 ± 0.004 ^B^	0.1243 ± 0.0068 ^B^	0.2274 ± 0.0215 ^A^	0.1943 ± 0.029 ^A^
21	0.1343 ± 0.0168 ^B^	0.1246 ± 0.0058 ^B^	0.1338 ± 0.0147 ^B^	0.3765 ± 0.0513 ^A^	0.3294 ± 0.033 ^A^
28	0.1429 ± 0.0222 ^B^	0.1499 ± 0.0088 ^B^	0.1344 ± 0.0156 ^B^	0.427 ± 0.0493 ^A^	0.3849 ± 0.0247 ^A^
35	0.1541 ± 0.0169 ^B^	0.176 ± 0.0259 ^B^	0.1487 ± 0.0157 ^B^	0.5265 ± 0.021 ^A^	0.4766 ± 0.0314 ^A^

^a^ Same superscript lowercase letters in the same row show no significant differences (*p* > 0.05). ^A,B^ Different superscript capital letters in the same row show extremely significant differences (*p* < 0.01).

**Table 2:** The OD_450_ of SIgA levels before and after immunizations.

Time			Group		
(d)	A	B	C	D	E
0	0.1096 ± 0.0098 ^a^	0.1091 ± 0.0051 ^a^	0.1127 ± 0.0086 ^a^	0.1114 ± 0.005 ^a^	0.1149 ± 0.0045 ^a^
14	0.1208 ± 0.0091 ^B^	0.1328 ± 0.0099 ^B^	0.1252 ± 0.0082 ^B^	0.2285 ± 0.0311 ^A^	0.1839 ± 0.0105 ^A^
21	0.1297 ± 0.0035 ^B^	0.1428 ± 0.0086 ^B^	0.1402 ± 0.0108 ^B^	0.2628 ± 0.0048 ^Aa^	0.1942 ± 0.041 ^Ab^
28	0.1595 ± 0.0182 ^B^	0.1613 ± 0.0167 ^B^	0.1515 ± 0.011 ^B^	0.34 ± 0.0415 ^A^	0.3024 ± 0.0858 ^A^
35	0.1855 ± 0.0161 ^B^	0.1981 ± 0.0238 ^B^	0.188 ± 0.0111 ^B^	0.4521 ± 0.0195 ^Aa^	0.3295 ± 0.032 ^Ab^

^a^ Same superscript lowercase letters in the same row show no significant differences (*p* > 0.05). ^A,B^ Different superscript capital letters in the same row show extremely significant differences (*p* < 0.01). ^Aa,Ab^ In the same row, the first capital letter (^A^) shows extremely significant differences in all groups (*p* < 0.01), and the second lowercase letter (^a,b^) show significant differences between group D and E (*p* < 0.05).

**Table 3:** Plasmids and primers sequences.

Strains, Plasmids, and Primer Sequences	Description	Reference or Restriction Site
Strains		
*Porcine rotavirus*	Strain G5P[7]	Vico Biology
*E. coli* DH5α and Rosetta (DE3)	Type strain	Tiangen
*B. subtilis* 168	Type strain	Bacillus Genetic Stock Center (BGSC)
*B. subtilis* BV	*B. subtilis* 168 amyE: CotB-VP8*, *Cm* ^r^	This work
Plasmids		
pET32a-VP8*	prokaryotic expression vector	Our lab
pDG364	*E.*-*coli*–*B.*-*subtilis* shuttle vector	BGSC
pDG364-VP8*	pDG364 derivative carrying the VP8* gene	This work
pDG364-CotB-VP8*	pDG364 derivative carrying the fusion *CotB*-VP8* gene	This work
Primer sequences		
VP8*-F ^a^	5′-CGCAAGCTTATGGCTTCGCTCATTTA-3′	*Hind I*
VP8*-R ^b^	5′-CGGGAATTCTTATCTTGTGTGCACTATCTC-3′	*EcoR I*
CotB-F	5′-CGGGATCCACGGATTAGGCCGTTTGTCC-3′	*BamH I*
CotB-R	5′-GGGAAGCTTGGATGATTGATCATCTGAAG-3′	*Hind III*
amyE-F	5′-CCAATGAGGTTAAGAGTATTCC-3′	null
amyE-R	5′-CGAGAAGCTATCACCGCCCAGC-3′	null

^a^ Forward, ^b^ Reverse. The underline indicates the restriction site.

**Table 4:** Program for the vaccination experiment and time of sample collection.

Groups	Strains	Method of Administration	Dose	Time of Administration	Time of Sample Collection
A	-	-	-	-	Days 0, 14, 21, 28, and 35
B	*B. subtilis* 168	Fed the mixed diet	5.0 × 10^6^ CFU/g	The whole period	Days 0, 14, 21, 28, and 35
C	*B. subtilis* 168	Gavage	2.0 × 10^10^ CFU/mL	Days 1–3, 14–16, and 28–30	Days 0, 14, 21, 28, and 35
D	*B. subtilis* BV	Fed the mixed diet	5.0 × 10^6^ CFU/g	The whole period	Days 0, 14, 21, 28, and 35
E	*B. subtilis* BV	Gavage	2.0 × 10^10^ CFU/mL	Days 1–3, 14–16, and 28–30	Days 0, 14, 21, 28, and 35
